# Polymer Capsules with Tunable Shell Thickness Synthesized via Janus-to-core shell Transition of Biphasic Droplets Produced in a Microfluidic Flow-Focusing Device

**DOI:** 10.1038/s41598-020-61641-8

**Published:** 2020-03-12

**Authors:** Siyuan Xu, Takasi Nisisako

**Affiliations:** 1 Department of Mechanical Engineering, School of Engineering, Tokyo Institute of Technology, Tokyo, Japan; 2 Laboratory for Future Interdisciplinary Research of Science and Technology (FIRST), Institute of Innovative Research, Tokyo Institute of Technology, R2-9, 4259 Nagatsuta-cho, Midori-ku, Yokohama, Kanagawa 226-8503 Japan

**Keywords:** Chemical engineering, Mechanical engineering, Materials science, Chemical engineering

## Abstract

Droplet microfluidics has enabled the synthesis of polymeric particles with controlled sizes, shell thickness, and morphologies. Here, we report the Janus to core-shell structural evolution of biphasic droplets formed in a microfluidic flow-focusing device (MFFD) for the synthesis of polymer microcapsules with oil core/thickness-tunable shell via off-chip photo- and thermally induced polymerization. First, nanoliter-sized biphasic Janus droplets comprising an acrylate monomer and silicone oil were generated in a co-flowing aqueous polyvinyl alcohol (PVA) solution in an MFFD on a glass chip. Immediately following their break-off, the produced Janus droplets started to change their geometry from Janus to core-shell structure comprising a single silicone-oil core and an acrylate-monomer shell by the minimization of interfacial energy. Thus, we could produce monodisperse core-shell drops with average diameters of 105–325 μm, coefficient of variation (CV) values of 1.0–4.5%, and shell thickness of 1–67 μm. Subsequently, these drops were synthesized to fabricate polymeric microcapsules with tunable shell thickness via photo- and thermally induced polymerization. By increasing the concentration of the photo- and thermal initiator, we successfully produced thinner and ultra-thin shell (800 nm thickness) microcapsules. The surface structure of resulting particles was smooth in photopolymerization and porous in thermal polymerization.

## Introduction

Microcapsules with core-shell structures, in which the active substances in cores are protected by the shells from the outer environment, have been applied in numerous fields such as foods^1,2^, cosmetics^3,4^, pharmaceutics^5^, printing^6,7^, and self-healing materials^8,9^. Microcapsules can be synthesized via a variety of techniques including spray drying^10^, layer-by-layer deposition^11^, interfacial polymerization^12^, coacervation^13^, or membrane emulsification^14^. Although these techniques can provide a high throughput, it remains difficult to fabricate uniform microcapsules with controlled size and high encapsulation efficiency.

Recently, droplet microfluidics has been shown to provide a new and promising route to synthesize microcapsules. Using microfluidic technology, one can easily produce monodisperse core-shell droplets as ideal templates for fabricating microcapsules via subsequent various solidification methods; examples of the solidification methods include photo or thermally induced free-radical polymerization^15,16^, solvent evaporation^17^, freezing^18^, and ironic cross-linking^19^. So far, the core-shell templates were prepared in two-step or one-step droplet formation. In the two-step method, using two T-junctions^20,21^, two flow-focusing junctions^22,23^, two co-flowing junctions^24^, or three-dimensional devices^25^, core drops are generated at first and then shell drops encapsulating these core drops are produced in a continuous phase as a separate step. Using this two-step approach, for example, acrylic capsules with aqueous cores with diameters of 10–340 µm and CVs ~5% were synthesized via the photopolymerization of water-in-oil-in-water (W/O/W)^26^ and water-in-oil-in-oil (W/O/O)^15,27^ droplets. Meanwhile, ethyl cellulose capsules with aqueous core (average diameters of 137 µm and CVs of 6.26%) and porous microcapsules composed of ethyl cellulose shell and poly(N-isopropylacrylamide) (PNIPAM) core (average diameter of 292 µm and CV of 6.3%) were prepared from W/O/W^28^ and W/(O/W)/W^29^ double emulsions via solvent evaporation. However, a limitation of this two-step formation method is that the flow rates of the external, middle, and inner phases need to be controlled precisely to generate the monodisperse core-shell droplets.

Compared to the two-step droplet formation method using two sequential emulsification steps, the one-step formation method only uses a single step to generate core-shell droplets in a cross-flowing junction^30,31^, and three-dimensional devices^32,33^; thus, providing an effective way to generate core-shell droplets with tunable shell thickness. For instance, using 1,6-hexanediol diacrylate (HDDA) as a polymerizable phase, filled microcapsules (diameters of 70–250 μm and shell thicknesses of 7–50 μm)^34^ and hollow core-shell microspheres with thin shells (10 μm thickness)^35^ were prepared from monodisperse W/O/W and gas-in-oil-in-water (G/O/W) droplets, respectively, via photo-induced polymerization. Moreover, polymer microcapsules with ultra-thin shells that can retain an extremely high-volume fraction of encapsulated actives were synthesized recently. For example, using solvent evaporation, biodegradable polymer microcapsules^36^ with poly (lactic acid) ultra-thin shell (80 nm thickness) and stimulus-responsive polystyrene capsules^37^ with ultra-thin shell (200 nm thickness) were synthesized from W/O/W droplets with diameters of 115–165 μm and CVs of 1.0–1.9%. Besides, photonic capsules composed of a water core with 100 mOsm/L and ETPTA ultra-thin shell (750 nm thickness) were fabricated via photo-induced polymerization^38^. However, few studies have mentioned thermally induced polymerization method to synthesize polymeric microcapsules with ultra-thin shells, which we believe is a more suitable method for many industrial applications.

Previously, we reported in the one-step formation of Janus droplets in a T-junction microfluidic device, the produced Janus droplets would change their structure to core-shell droplets at equilibrium due to the minimization of interfacial energy among the three liquid phases. Subsequently, the resultant core-shell drops can be used as templates for the fabrication of polymeric capsules with tunable shell thickness via off-chip photo-induced polymerization^31^. To the best of our knowledge, however, the Janus-to-core-shell transition of biphasic droplets have not been reported using a microfluidic flow-focusing device (MFFD).

Here, we report a novel microfluidic approach to synthesize polymeric microcapsules with tunable shell thickness and surface morphology via off-chip photo- and thermally induced polymerization. We fabricated the particles from the biphasic Janus droplets generated in an MFFD, in which droplets underwent a structural evolution from Janus to core-shell droplets comprising an oil core and a curable monomer shell at equilibrium. The size and shell thickness of drops could be tuned precisely and flexibly, which enabled the fabrication of microcapsules with tunable shell thickness. The key to the successful synthesis of ultra-thin shell polymeric microcapsules was to increase the initiator concentration. In addition, we demonstrated the effect of solidification methods on surface morphology of microcapsules.

## Methods

### Device fabrication and assembly

**Figure 1:**
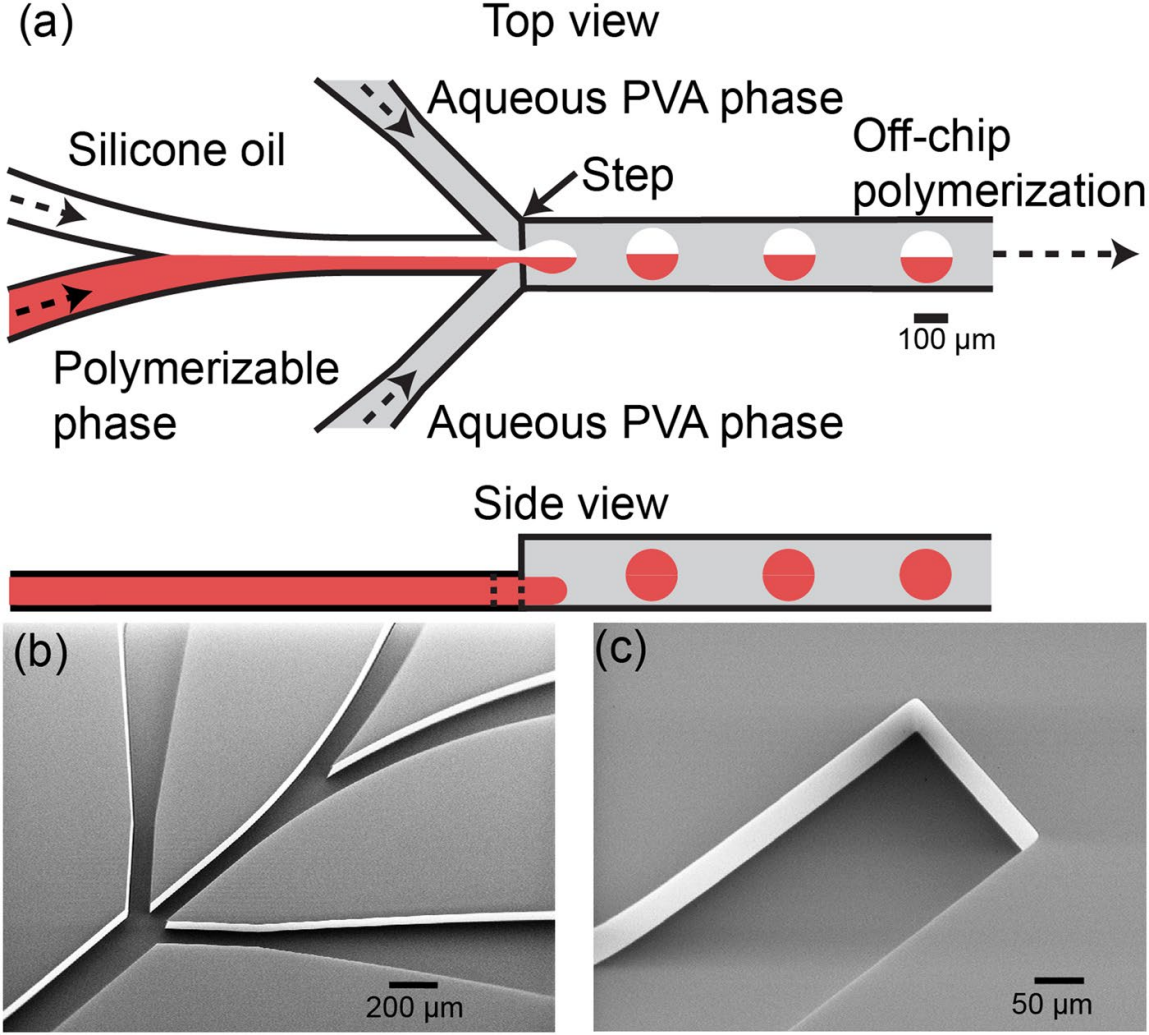
Microfluidic channels for generating biphasic droplets. (**a**) Schematic illustration of a microfluidic biphasic droplet generator with a deeper drainage region. (**b**,**c**) SEM images of (**b**) a flow-focusing geometry and (**c**) a microgroove for the deeper drainage channel fabricated on two separate glass chips.

A microfluidic Janus droplet generator that has channels with a rectangular cross-sectional shape was prepared on a glass chip (15 mm × 15 mm × 3.5 mm) using deep reactive ion etching^39,40^. The Y-shaped channel for infusing two organic phases and the two co-flowing channels for infusing aqueous phases have a depth of 100 μm and a width of 100 μm around the sheath-focusing junction. The drainage channel has a depth of 200 μm and a width of 200 μm (Fig. 1a). This deeper drainage region was prepared by fusion bonding of two separate chips each with microfabricated grooves (depth 100 μm) with precise alignment (Fig. 1b,c).

The prepared glass chip was assembled with a stainless-steel holder (36 mm × 36 mm × 10 mm) that comprised a supporting layer and a cover with a square-hole. The glass chip was placed into the supporting layer that has threaded holes to connect the poly(tetrafluoroethylene) (PTFE) tubes (0.8 mm internal diameter) for the transportation of fluids. The cover with a square-hole was used to fasten the glass chip, thus achieving direct on-chip optical microscopy inside the channels.

### Materials

We used an acrylate monomer (1, 6-hexanediol diacrylate (HDDA), dynamic viscosity *η*_m_ = 6.35 mPa s, density *ρ*_m_ = 1.02 g cm^−3^, Shin-Nakamura Kagaku, Japan) as a polymerizable phase, and silicone oil (SH200–10, 20, 50, 100CS, Dow Corning Toray, Japan) as a non-polymerizable phase. Unless otherwise specified, the viscosity of silicone oil is 10 cSt. A 2.0 wt % aqueous polyvinyl alcohol (PVA, 87–89% hydrolyzed) solution was used as the continuous phase. For subsequently polymerization, a photoinitiator (2.0–10.0 wt %, Darocur 1173, Ciba Japan K. K., Japan) or thermal initiator (2.0–15.0 wt %, V-65, Fujifilm Wako Pure Chemical Corporation, Japan) was added in HDDA to generate a mixture. An oil soluble dye (Oil red O, Sigma-Aldrich, MO, USA) was dissolved in HDDA to differentiate the two organic phases visually. The liquid viscosities and interfacial tensions were adapted from ref. ^31^.

### Preparation of polymer microcapsules

Polymer microcapsules were prepared by solidifying generated droplets via an off-chip photo or thermally induced polymerization method. For photo-induced polymerization, the generated droplets were collected in a beaker and continuously polymerized by an ultra-violet (UV) light source (LA-410UV, Hayashi, Japan). The distance for irradiation from the UV light source was about 15–20 cm. For thermally induced polymerization, the generated droplets were guided into a plastic cup containing the PVA solution directly via the PTFE tube. The plastic cup was placed in a beaker containing pure water and heated to 80–85 °C, monitored by a thermometer using a ceramic hot stirrer (CHPS-170DN, AS ONE Corporation, Japan).

A nylon mesh sheet (grid size: 42 μm × 42 μm) was used to remove smaller satellite microcapsules by filtering the synthesized microcapsules. Then, acetone and ethanol were used to wash microcapsules on the mesh sheet slightly.

### Measurement and peripheral equipment

Liquids were filled in glass syringes (1000 series, Hamilton Company, USA) and supplied to the microfluidic device using syringe pumps (KDS200, KD Scientific, USA). An optical microscope (BX-51, Olympus, Japan) equipped with a high-speed video camera (Fastcam Mini AX50, Photron, Japan) was used for the observation of droplet generation. Software ImageJ (National Institutes of Health, NY, USA) was used to measure the diameters of droplets.

### Characterization of microcapsules

A scanning electron microscope (SEM, JSM-6610LA, JEOL, Japan) was used to observe the surface and structure of the microcapsules. For preparation of the cross-sections of microcapsules, they were dispersed randomly in epoxy resin and sliced manually using a razor blade.

## Results and Discussion

### Generation of biphasic janus droplets

**Figure 2:**
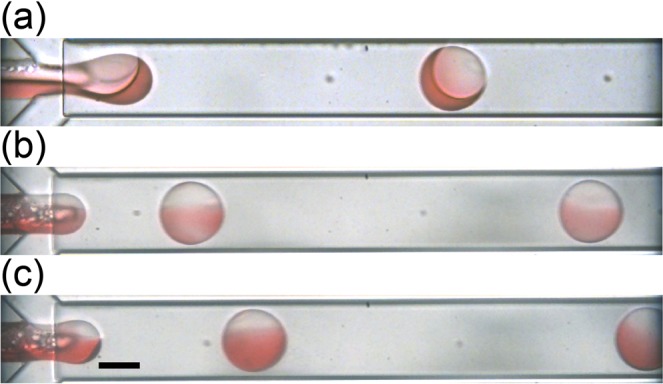
Formation of Janus droplets at different flow rate ratios of acrylate monomer (*Q*_m_) and silicone oil (*Q*_s_). (**a**) *Q*_m_:*Q*_s_ = 1:1. (**b**) *Q*_m_:*Q*_s_ = 1:9, (**c**) 1:4. Total flow rate of two organic phases (*Q*_d, total_ = *Q*_m_ + *Q*_s_) is 1.0 mL h^−1^, and the flow rate of aqueous PVA phase (*Q*_c_) is 6.0 mL h^−1^ × 2. Scale bar: 100 μm.

Biphasic droplets with uniform sizes were generated when we controlled the flow rates of aqueous and two organic phases at a low capillary number and Reynolds number regions. Figure 2a shows the formation of biphasic droplets when the flow rates of HDDA (*Q*_m_), silicone oil (*Q*_s_), and PVA solution (*Q*_c_) stream were 0.5 mL h^−1^, 0.5 mL h^−1^, and 12.0 mL h^−1^, respectively. At the flow-focusing junction, two immiscible parallel organic phases were sheared into nanoliter-sized biphasic droplets at the dripping regime in 8 ms by the aqueous PVA phase. However, it was difficult to confirm whether the silicone oil segment was partially engulfed or fully engulfed by the HDDA segment at *Q*_m_:*Q*_s_ = 1:1. In contrast, by observing the structure of the biphasic droplet generated right after the junction at *Q*_m_:*Q*_s_ = 1:9 (Fig. 2b), we could confirm that both HDDA and silicone oil parts were partially exposed to the PVA solution. Thus, we confirmed the formation of Janus droplets comprised of a curable HDDA segment and a non-curable silicone oil segment. Then, the Janus droplets kept their morphology with silicone oil gradually engulfed by the HDDA segment, flowing downstream to the outlet. By tuning the *Q*_m_/*Q*_s_ ratio, we could generate biphasic droplets with different volume ratios. For example, formation of the Janus droplets at the *Q*_m_/*Q*_s_ ratio of 1/4 is shown in Fig. 2c (see supplementary videos).

For the generation of Janus droplets comprised of two immiscible segments in this study, it is well known that the mixing of two segments will not occur^40^. However, we could observe the phenomenon of color spreading from HDDA to the silicone oil segment when the HDDA containing oil soluble dye was used. We consider the color spreading phenomenon was because the HDDA layer was forming on the silicone oil segment.

In our previous study^31^, we realized the generation of Janus droplets using the asymmetric T-shaped microfluidic device. In this study, we confirmed that a symmetric MFFD was also feasible to generate Janus droplets comprised of the same segments, indicating that the difference in microfluidic geometries would not affect the droplet structure. This similarity is presumably because the interfacial energy, rather than the hydrodynamic effects, is playing a dominant role in the phase separation within the forming droplets. Meanwhile, MFFD has the advantage of providing a more stable way to generate droplets with controlled sizes in dripping regime. This is because the disperse phase in MFFD is surrounded by two symmetric continuous-phase streams and does not wet the sidewalls^41,42^, while the disperse phase in a T-junction device stays in contact with one of the sidewalls and eventually wets the sidewall to affect the formation of droplets^43^.

### Effect of silicone oil viscosity and channel geometry on droplet formation

**Figure 3:**
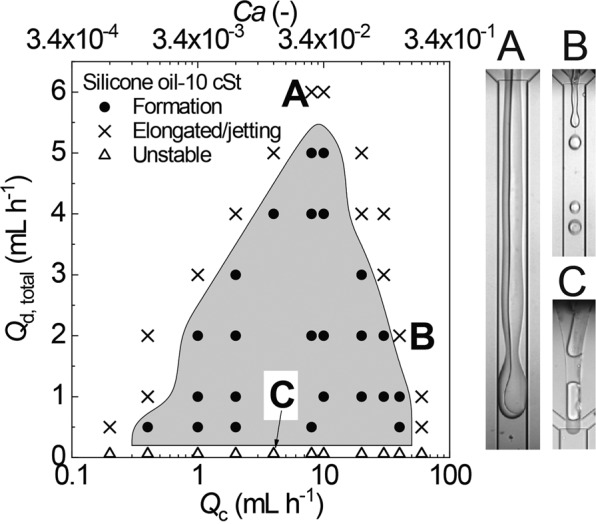
A flow pattern diagram showing the condition for the formation of biphasic droplets with 10 cSt silicone oil. The solid circles represent the condition where monodisperse biphasic droplets can form. The crosses represent the condition of elongated steam (inset A) or irregular jetting regime (inset B). The open triangles represent the condition where two-phase stream of acrylate monomer and silicone oil becomes unstable (inset C). *Ca* = *ηνγ*^−1^, where *η* = 1.95 mPa s is dynamic viscosity of the aqueous PVA solution, *ν* is the mean velocity of the aqueous PVA stream, and *γ* = 5.95 mN m^−1^ is average interfacial tension of HDDA/PVA interface and silicone oil/PVA interface.

One of the critical parameters in microfluidic droplet formation is fluid viscosity. For example, the study about the formation of oil-in-water droplets in the MFFD showed that highly viscous disperse phase produced larger droplets at the same flow rate ratios^44^. By varying the viscosity of the silicone oil at 10, 20, 50 and 100 cSt, we analyzed the flow conditions necessary to generate biphasic droplets at the junction when we kept *Q*_m_:*Q*_s_ = 1 and changed *Q*_c_. It is clear that the flow diagrams have an enclosed mountain-shape^45^ with capillary number (*Ca*) ranging from 10^−3^ to 10^−1^ (Figs. 3 and S1). The flow diagrams were dependent on the silicone oil viscosity. With an increase in silicone oil viscosity, the maximum flow rate of the two organic phases capable of producing biphasic droplets reduced gradually, which caused the region of monodisperse droplets to become smaller. The results were consistent with the previous study of generating oil-in-water droplets in the MFFD, where for the disperse phase with low viscosity, the dripping flow regime covered a wider range than the disperse phase with high viscosity^46^.

**Figure 4:**
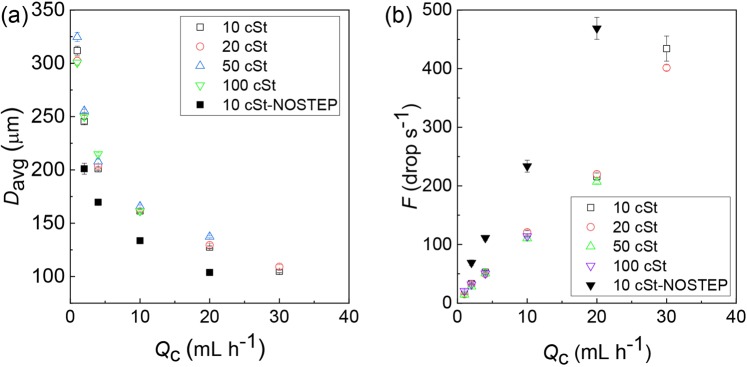
Continuous phase flow rate vs. droplet size and generation frequency measured with silicone oils of different viscosities and two different flow-focusing chips. (**a)** The continuous phase flow rate *Q*_c_ vs. average diameter of the biphasic droplets *D*_avg_. (**b**) The *Q*_c_ vs. generation frequency_._ *Q*_m_/*Q*_s_ = 1. *Q*_d, total_ = 1.0 mL h^−1^.

Then, we analyzed the effect of silicone oil viscosity and channel size on the diameter (*D*_avg_) and break-off frequency (*F*) of biphasic droplets by tuning *Q*_c_ with a fixed *Q*_m_ and *Q*_s_. As shown in Fig. 4, with the same device, the difference in viscosities of silicone oils did not have a significant impact on both *D*_avg_ and *F*. The increase of *Q*_c_ from 1.0 to 30.0 mL h^−1^ resulted in the decrease of *D*_avg_ from 325 to 105 μm with the CVs ranging from 1.0% to 4.5% (Fig. 4a); *F* and *Q*_c_ had a proportionality relationship and increased linearly from 14 to 434 droplets per second (Fig. 4b). This experimental result agreed well with the law discussed in ref. ^47^, where shear-driven breakup mechanism indicates that *D*_avg_ ∝ *Q*_c_^−1/3^, leading to the proportionality relation *F* ∝ *Q*_c_. Meanwhile, in contrast to the silicone oil viscosities, the channel size had a significant effect on *F* and *D*_avg_. When the droplets were produced in an MFFD without a step^40,45^, *F* became ~2.2 times higher and *D*_avg_ was reduced by 20% at a fixed *Q*_c_.

### Core-shell droplets evolved from janus droplets

First, the generated biphasic droplets that flowed out the device through a 5-cm-long PTFE drain tube were collected on a glass slide, and their structure was immediately locked via photopolymerization. Then, as shown in Fig. S2a, we observed that both spherical and non-spherical particles were present, which suggests the droplets were undergoing a morphological transition process, and they had not reached the equilibrium state yet within the residence time in the microfluidic device (~9 s).

Next, we increased the length of the drainage tube to increase the residence time. When we used a 150-cm-long drain tube, the morphology became consistent, which suggests the morphology of droplets reached the equilibrium state (Fig. S2b).

**Figure 5:**
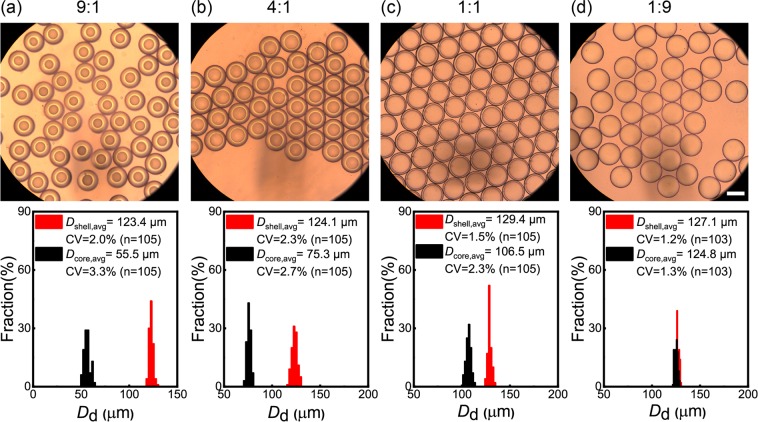
Monodisperse core-shell droplets and their size distributions obtained at different *Q*_m_/*Q*_s_ ratios. (**a**) *Q*_m_: *Q*_s_ = 9:1, (**b**) 4:1, (**c**) 1:1, and (**d**) 1:9. *Q*_d, total_ = 1.0 mL h^−1^. *Q*_c_ = 10.0 mL h^−1^ × 2. Scale bar: 100 μm.

Figure 5 shows the bright field micrographs of the droplets and their size distributions at different *Q*_m_/*Q*_s_ ratios, when we kept total flow rate of two organic phases (*Q*_d, total_ = *Q*_m_ + *Q*_s_) and *Q*_c_ at 1.0 mL h^−1^ and 20.0 mL h^−1^, respectively. The observation was performed immediately following the collection using the 150-cm-long drain tube. The uniform appearance indicated that droplets reached the equilibrium state. Our previous study described that the morphology of biphasic droplets comprised of the same materials at equilibrium was core-shell state with a silicone oil core and a monomer shell, due to the minimization of interfacial energy^31^. This reflected the morphology transition from Janus-to-core shell geometry was complete. As *Q*_m_/*Q*_s_ decreased from 9/1 to 1/9, shell diameters varied from 123 μm to 129 μm, and the core diameters increased from 56 μm to 125 μm, leading to the interfaces of the cores being closer to those of the shells. In all conditions, the core-shell droplets had uniform sizes with the CVs at the range of 1.3% to 3.3% for cores, and 1.2% to 2.3% for shells.

**Figure 6:**
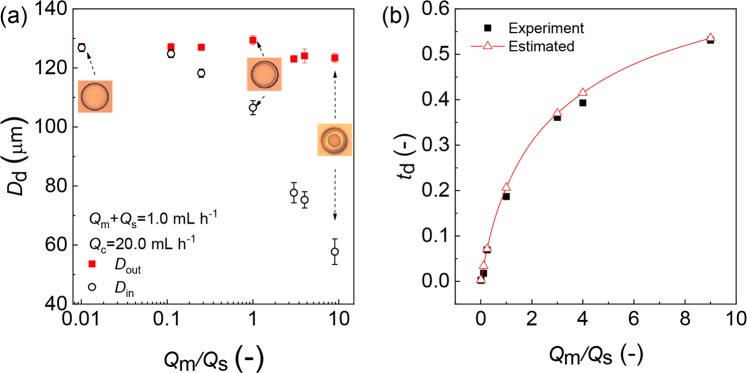
Core-shell droplets with varying shell thickness. (**a**) The inner (*D*_in_) and outer (*D*_out_) diameters of core-shell droplets. (**b**) Effect of *Q*_m_/*Q*_s_ on relative shell thickness *t*_d_. The solid line represents the estimation from Eq. (1). The *t*_d_ varies from 0.3% to 53% with the increase of *Q*_m_/*Q*_s_ from 1/99 to 9/1. *Q*_d, total_ = 1.0 mL h^−1^, *Q*_c_ = 10.0 mL h^−1^ × 2.

The relationship between *Q*_m_/*Q*_s_ and inner and outer diameters of the core-shell droplets is shown at Fig. 6a. It can be seen that with an increase in *Q*_m_/*Q*_s_ from 1/99 to 9/1, the outer diameter (*D*_out_) was 125 ± 4 μm without a significant change, while the inner diameter (*D*_in_) decreased from 127 μm to 56 μm. In this condition, the difference in size between cores and shells varied from 1 μm to 67 μm. The results indicated that we could precisely control the shell thickness of the core-shell droplets by keeping *Q*_d, total_ and *Q*_c_ constant and varying *Q*_m_/*Q*_s_. We define the relative shell thickness of the core-shell droplets (*t*_d_) as

1 $${t}_{{\rm{d}}}=\frac{{D}_{{\rm{out}}}-{D}_{{\rm{in}}}}{{D}_{{\rm{out}}}}=1-{\left(1+\frac{{Q}_{{\rm{m}}}}{{Q}_{{\rm{s}}}}\right)}^{-1/3}$$

Figure 6b shows the comparison between experimental and estimated results of *t*_d_. The estimated *t*_d_ decreased from 53% to 0.3% by varying *Q*_m_/*Q*_s_ from 9/1 to 1/99. The slight difference between the experimental and estimated results can be explained by the different values of refractive indices of light in the HDDA (*n* = 1.456) and the PVA aqueous phases (*n* ~ 1.33), which caused a slight magnification of the cores. This slight discrepancy was also found in the core-shell droplets generated from the same materials at T-junction, where *t*_d_ ranged from 30% to 0.4%, and the experiment results were slightly smaller than the estimated ones^31^.

### Photo- and thermal polymerization of microcapsules and their characterization

**Figure 7:**
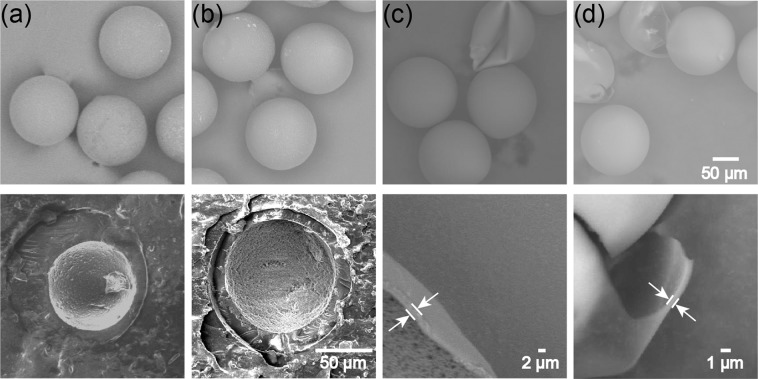
Surface morphology (top), cross-section and shell thickness (bottom) of photo-polymerized capsules. (**a**) Particles prepared with *Q*_m_:*Q*_s_ = 3:1 and 2.0 wt % initiator concentration. (**b**) Particles prepared with *Q*_m_:*Q*_s_ = 1:1 and 2.0 wt % initiator concentration. (**c**) Particles with thin shell prepared with *Q*_m_:*Q*_s_ = 1:9 and 5.0 wt % initiator concentration. (**d**) Particles with ultra-thin shell prepared with *Q*_m_:*Q*_s_ = 1:99 and 10.0 wt % initiator concentration. All particles were produced at *Q*_d, total_ = 1.0 mL h^−1^, *Q*_c_ = 8.0 mL h^−1^ × 2.

For off-chip photopolymerization, we collected the core-shell droplets at different *Q*_m_/*Q*_s_ ratios and exposed them under UV light. The SEM observation of the cross-linked particles and their cross-sections revealed that microcapsules with smooth surfaces could be obtained when *Q*_m_/*Q*_s_ = 3/1 (Fig. 7a), and 1/1 (Fig. 7b), respectively, with 2.0 wt % photo initiator concentration. However, the thinner shells of core-shell droplets generated at lower *Q*_m_/*Q*_s_ ratio of 1/9 here could not be polymerized with the same 2.0 wt % photo initiator concentration. This insufficient polymerization phenomenon was also observed in our previous study with T-junction device. Although microcapsules could be produced at *Q*_m_/*Q*_s_ = 3/1, 1/1, 1/7, the microcapsules with thinner shells at *Q*_m_/*Q*_s_ = 1/79 could not be fabricated when initiator concentration was fixed at 1.0 wt %^31^. We considered one possible reason to be the outward diffusion of the photo-initiator in the shell.

In this study, we could fabricate microcapsules with thinner and ultra-thin shells simply by increasing the initiator concentration. By increasing the concentration from 2.0 to 5.0 wt %, microcapsules with thinner shells (2 μm thickness) could be fabricated when *Q*_m_/*Q*_s_ = 1/9 (Fig. 7c). Moreover, microcapsules with ultra-thin shells (800 nm thickness) could be fabricated experimentally when *Q*_m_/*Q*_s_ dropped to 1/99 with 10.0 wt % photo initiator concentration (Fig. 7d). The experimental shell thicknesses were close to the estimated results calculated using abovementioned Eq. (1).

**Figure 8:**
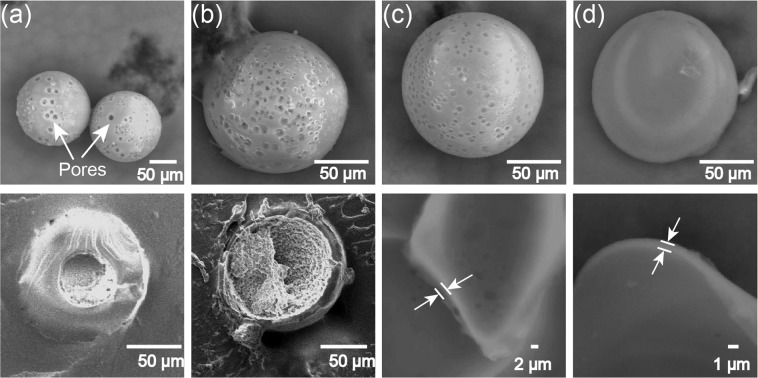
Surface morphology (top), cross-section and shell thickness (bottom) of thermally polymerized capsules. (**a**) Particles prepared with *Q*_m_:*Q*_s_ = 9:1 and 2.0 wt % initiator concentration. (**b**) Particles prepared with *Q*_m_:*Q*_s_ = 1:1 and 2.0 wt % initiator concentration. (**c**) Particles with thin shell prepared with *Q*_m_:*Q*_s_ = 1:9 and 5.0 wt % initiator concentration. (**d**) Particles with ultra-thin shell prepared with *Q*_m_:*Q*_s_ = 1:99 and 15.0 wt % initiator concentration. All particles were produced at *Q*_d, total_ = 1.0 mL h^−1^, *Q*_c_ = 8.0 mL h^−1^ × 2.

For off-chip thermally induced polymerization, we collected the core-shell droplets at different *Q*_m_/*Q*_s_ ratios and guided them into a heated PVA aqueous solution. The SEM observation of the particles prepared at *Q*_m_:*Q*_s_ = 9:1 with 2.0 wt % thermal initiator concentration revealed that they were indeed microcapsules. However, we found that the capsules had many pores on their surfaces (Fig. 8a), in contrast to the smooth surfaces of the photopolymerized capsules. Similarly, microcapsules could be obtained when *Q*_m_/*Q*_s_ = 1/1 with 2.0 wt % thermal initiator concentration (Fig. 8b). However, the thinner shell of core-shell droplets generated at lower *Q*_m_/*Q*_s_ ratio of 1/9 could not be polymerized with the same initiator concentration (2.0 wt %), possibly due to the outward diffusion of the thermal-initiator in the shell. By increasing the concentration from 2.0 wt % to 5.0 wt %, microcapsules with thinner shells (2 μm thickness) could be fabricated when *Q*_m_/*Q*_s_ = 1/9 (Fig. 8c). Moreover, microcapsules with ultra-shells (800 nm thickness) could be fabricated when *Q*_m_/*Q*_s_ = 1/99 with 15.0 wt % thermal initiator concentration (Fig. 8d). We also confirmed the surfaces of the microcapsules became smoother when the shells became thinner (*Q*_m_/*Q*_s_ decreased from 9/1 to 1/99).

The porous surface morphology of microcapsules was presumable because the Nitrogen−Nitrogen double bond cleavage of the initiator V-65 in HDDA shell could produce nitrogen during the thermally induced polymerization. When *Q*_m_/*Q*_s_ = 9/1, we consider the shells were thick enough to enable the accumulation and growth of nitrogen bubbles, which caused formation of pores on the shells during the solidification. However, with the decrease of volume fraction of HDDA (*Q*_m_/*Q*_s_ decrease from 9/1 to 1/99), the shells became thinner, and then the produced nitrogen was released directly without accumulation, which resulted in the smoother surfaces.

## Conclusion

We demonstrated the ability of a microfluidic flow-focusing device to generate biphasic Janus droplets and subsequent transformation to core-shell droplets for the synthesis of polymer microcapsules via photo- and thermally induced polymerization. The shell thickness of the droplets and resultant polymer microcapsules could be tuned by changing the flow rate ratios of the acrylate monomer and silicone oil phases. The novelty of this synthesis lies in the fact that polymeric microcapsules with ultra-thin shell thickness of 800 nm were successfully synthesized experimentally by increasing photo- and thermal initiator concentration. Further, compared with smooth surfaces of photopolymerized capsules, thermally induced polymerization could produce polymer microcapsules with porous surface morphology. From our previous work with the T-junction device^31^, we consider that the change in device geometry does not have a significant impact on the availability of this synthetic process. Nevertheless, the MFFD in the present study might be a better choice since it can provide more stable droplet generation. We believe the present simple technology could be applied to the synthesis of microcapsules with desired functionality for target applications, e.g., in cosmetics, foods and drug encapsulation.

## Supplementary information

Supplementary Information.

Supplementary Information2.

Supplementary Information3.

Supplementary Information4.
