# Supplementary material for: Polymer Capsules with Tunable Shell Thickness Synthesized via Janus-to-core shell Transition of Biphasic Droplets Produced in a Microfluidic Flow-Focusing Device
Source: Sci Rep. 2020 Mar 12;10:4549. doi: 10.1038/s41598-020-61641-8 (PMC7067790; doi:10.1038/s41598-020-61641-8)
Supplement: Supplementary file 1 — Supplementary Information. [file 41598_2020_61641_MOESM1_ESM.pdf]

Polymer Capsules with Tunable Shell Thickness Synthesized via Janus-to-core shell Transition of Biphasic Droplets Produced in a Microfluidic Flow-Focusing Device

Siyuan Xu[a], *Takasi Nisisako*[b*]

[a] Department of Mechanical Engineering, School of Engineering, Tokyo Institute of Technology, Tokyo, Japan
[b] Laboratory for Future Interdisciplinary Research of Science and Technology (FIRST), Institute of Innovative Research, Tokyo Institute of Technology, R2-9, 4259 Nagatsuta-cho, Midori-ku, Yokohama, Kanagawa, 226-8503, Japan
*To whom correspondence should be addressed.

Electronic Supplementary Information

Email: nisisako.t.aa@m.titech.ac.jp

Supplementary figures

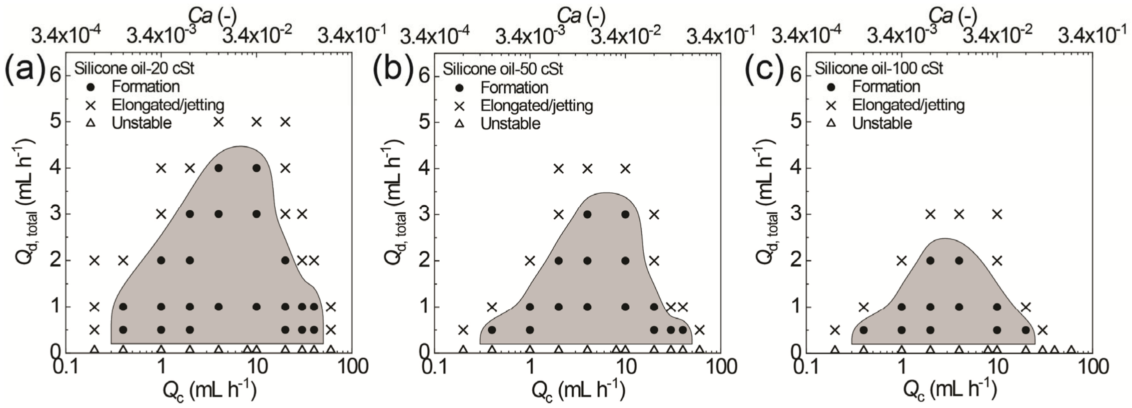

Figure S1 Flow pattern diagrams for producing biphasic droplets with different silicone oil viscosities: (a) 20 cSt; (b) 50 cSt; (c) 100 cSt.

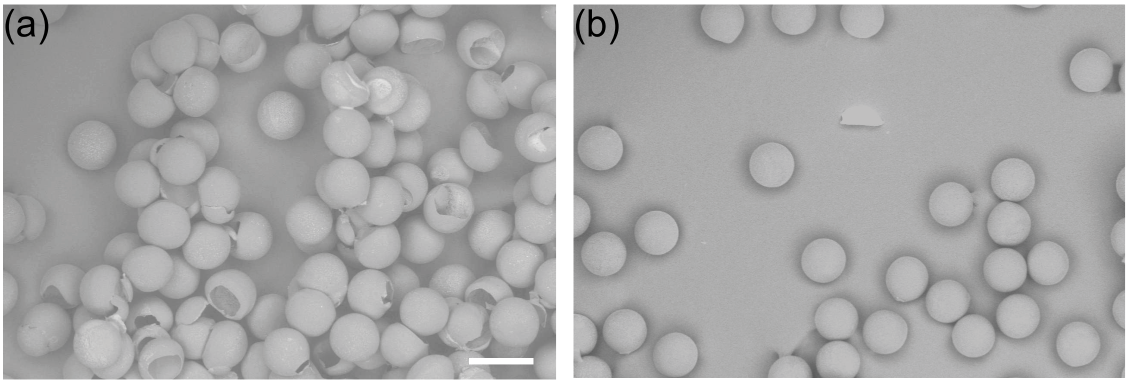

Figure S2 Particles obtained using different tube length (a) 5 cm, (b) 150 cm. Q_{m}:Q_{s} =1:1. $Q_{\mathrm{d,\,total}}$ = 1.0 mL h^{-1}, Q_{c} = 6.0 mL h^{-1} × 2. Scale bar: 200 μm.

Supplemental Movie Caption

FF_Janus01.mov: Movie clip of the formation of Janus droplets at a flow-focusing microfluidic geometry, recorded at 10,000 fps. Flow rates of monomer (Q_m) and silicone oil (Q_s) are $Q_m = Q_s = 0.5$ mL h^{-1}. Flow rate of PVA aqueous phase (Q_c) is $Q_c = 6.0$ mL h^{-1} × 2.

FF_Janus02.mov: Movie clip of the formation of Janus droplets at a flow-focusing microfluidic geometry, recorded at 10,000 fps. Flow rates are $Q_m = 0.1$ mL h^{-1}, $Q_s = 0.9$ mL h^{-1}, and $Q_c = 6.0$ mL h^{-1} × 2.

FF_Janus03.mov: Movie clip of the formation of Janus droplets at a flow-focusing microfluidic geometry, recorded at 10,000 fps. Flow rates are $Q_m =0.2$ mL h^{-1}, $Q_s = 0.8$ mL h^{-1}, and $Q_c = 6.0$ mL h^{-1} × 2.
